# Kinetics of Label Retaining Cells in the Developing Rat Kidneys

**DOI:** 10.1371/journal.pone.0144734

**Published:** 2015-12-09

**Authors:** Jianwen Wang, Guiting Lin, Amjad Alwaal, Xiaoyu Zhang, Guifang Wang, Xingyuan Jia, Lia Banie, Jacqueline Villalta, Ching-Shwun Lin, Tom F. Lue

**Affiliations:** 1 Department of Urology, Beijing ChaoYang Hospital, Capital Medical University, 8 Gongtinanlu, Beijing, 100020, China; 2 Knuppe Molecular Urology Laboratory, Department of Urology, School of Medicine, University of California San Francisco, San Francisco, CA, 94143-0738, United States of America; Center for Molecular Biotechnology, ITALY

## Abstract

**Background:**

The kidney is a specialized low-regenerative organ with several different types of cellular lineages. The BrdU label-retaining cell (LRCs) approach has been used as part of a strategy to identify tissue-specific stem cells in the kidney; however, because the complementary base pairing in double-stranded DNA blocks the access of the anti-BrdU antibody to BrdU subunits, the stem cell marker expression in BrdU-labeled cells are often difficult to detect. In this study, we introduced a new cell labeling and detection method in which BrdU was replaced with 5-ethynyl-2-deoxyuridine (EdU) and examined the time-dependent dynamic changes of EdU-labeled cells and potential stem/progenitor markers in the development of kidney.

**Methods:**

Newborn rats were intraperitoneally injected with EdU, and their kidneys were harvested respectively at different time points at 1 day, 3 days, 1 week, 2 weeks, and 6 weeks post-injection. The kidney tissues were processed for EdU and cellular markers by immunofluorescence staining.

**Results:**

At the early stage, LRCs labeled by EdU were 2176.0 ± 355.6 cells at day one in each renal tissue section, but dropped to 168 ± 48.4 cells by week 6. As time increased, the numbers of LRCs were differentially expressed in the renal cortex and papilla. At the postnatal day one, nearly twice as many cells in the cortex were EdU-labeled as compared to the papilla (28.6 ± 3.6% vs. 15.6 ± 3.4%, *P*<0.05), while there were more LRCs within the renal papilla since the postnatal week one, and at the postnatal week 6, one third as many cells in the cortex were EdU-labeled as compared to the papilla (2.5 ± 0.1% vs. 7.7 ± 2.7%, *P*<0.05). The long-term LRCs at 6-week time point were associated exclusively with the glomeruli in the cortex and the renal tubules in the papilla. At 6 weeks, the EdU-labeled LRCs combined with expression of CD34, RECA-1, Nestin, and Synaptopodin were discretely but widely distributed within the glomeruli; Stro-1 around the glomeruli; and α-smooth muscle actin (SMA) in arteries. Conversely, co-expression of CD34, RECA-1, and Nestin with the long term EdU-labeled LRCs was significantly lower in renal tubules (*P*<0.01), while Stro-1 and Synaptopodin were not detected.

**Conclusion:**

Our data found that at 6-week time point, EdU-labeled LRCs existing in the glomeruli expressed undifferentiated podocyte and endothelial markers at high rates, while those in the renal tubules expressed Nestin and vascular markers at low rates. To understand the characterization and localization of these EdU-LRCs, further studies will be needed to test cell lineage tracing, clonogenicity and differentiation potency, and the contributions to the regeneration of the kidney in response to renal injury/repair.

## Introduction

Emerging experimental evidence have shown that tissue-specific stem cells exist in most organs and tissues, and their self-renewal and multipotent properties make them support tissue homoeostasis by producing new distinct differentiated cells during the normal tissue cycling [1] and often respond dynamically to regenerate damaged tissue in times of injury [2,3]. On the other hand, it has also been proposed that tissue-specific stem cells are the seeds of tumors and thus potential treatment targets [4,5]. Therefore, the identification of tissue-specific stem cells is of great interest to both regenerative medicine and cancer therapy. However, in most instances, such as in the case with kidneys, the search for tissue-specific stem cells has proven to be difficult due to the lack of specific markers [1,3]. Recently, progress has been made, owing to studies that employed a combination of the “label-retaining cell (LRC)” strategy and semi-specific stem cell markers such as c-Kit, Sca-1, CD34, and CD133 [3].

The LRC strategy generally relies on the injection of thymidine analog 5-bromo-2-deoxyuridine (BrdU) into newborn animals, followed by the immunohistochemical detection of BrdU-labeled cells several weeks later [6,7]. Such BrdU+ cells, (i.e. LRCs), are believed to represent stem cells due to their ability to stay quiescent after a brief period of cellular division. However, BrdU labeling method has several limitations. First, the BrdU-labeled cells are often difficult to detect because of unremarkable color distinctions between BrdU and nuclear stains (brown vs. purplish brown). Another major disadvantage is that the complementary base pairing in double-stranded DNA blocks the access of the anti-BrdU antibody to BrdU subunits. To expose the epitope, tissue samples are subjected to harsh staining conditions (such as strong acids and high temperature) which invariably degrade the structure of cellular proteins and consequently make it difficult to determine stem cell marker expression in BrdU-labeled cells [8]. To overcome these difficulties, a new LRC procedure by the usage of 5-ethynyl-2-deoxyuridine (EdU) has been recently introduced [9]. In our previous studies, EdU is proven to fulfill this screening method for stem/progenitor cells and more importantly to overcome the ambiguity when double staining with cellular markers [10–12].

In this study, we injected EdU into newborn rats and examined the time-dependent distribution of EdU-labeled cells in the kidney. In particular, we investigated the co-expression of EdU-labeled retaining cells with various cell markers in the renal cortex and papilla tissues.

## Materials and Methods

### Experimental animals and EdU labeling

This study was carried out in strict accordance with the recommendations in the Guide for the Care and Use of Laboratory Animals of the National Institutes of Health. All animal experiments were approved by the Institutional Animal Care and Use Committee at School of Medicine, University of California, San Francisco. Pregnant Sprague–Dawley rats were purchased from Charles River Laboratories (Wilmington, MA), and then housed in an AAALAC-accredited facility with a 12-hour light-dark cycle and allowed water and food ad libitum. A total of 25 male neonatal pups delivered by these primiparous rats were used for this study.

We used the EdU retaining method to label and then identify/quantify LRCs in the developing rat kidney. As described in our previous studies [11], each pup received intraperitoneal injection of EdU (50 mg/kg, Invitrogen, Carlsbad, CA) immediately after birth. These EdU-injected rats were anesthetized (pentobarbital sodium 150 mg/kg, i.p.) and sacrificed at different time points at 1 day, 3 days, 1 week, 2 weeks, and 6 weeks post-injection, respectively. Five rats for each time point were used. The kidney tissues were harvested and then analyzed for EdU staining nuclei by immunohistochemistry and immunofluorescence.

### Preparation of tissue sections

Freshly dissected kidney tissue was fixed for 4 hours with cold 2% formaldehyde and 0.002% picric acid in 0.1 M phosphate buffer, followed by overnight immersion in buffer solution containing 30% sucrose. Tissues were frozen in optimum cutting temperature compound (Sakura Finetek, Torrance, CA), and stored at -80°C until use. Sections were cut at 5 μm, adhered to charged slides, and air dried for 5 minutes before staining.

### Immunofluorescence staining

Frozen tissue sections were placed in 0.3% H_2_O_2_/methanol for 10 min, washed twice in PBS for 5 min, and incubated with 3% horse serum in PBS/0.3% Triton X-100 for 30 min at room temperature. After draining the solution from the tissue section, the tissue was incubated with mouse anti-CD34 (SC-7324, Santa Cruz Biotech, Santa Cruz, CA), mouse anti-Synaptopodin (Q01246M, Meridian Life Science, Inc, Memphis, TN), rabbit anti–smooth muscle actin (SMA) (ab5694, Abcam, Inc., Cambridge, MA), mouse anti-Nestin (MAB353, EMD Millipore, Billerica, MA), mouse anti-RECA (MCA970R, AbD Serotec, Raleigh, NC), mouse anti-Stro-1 (MAB1038, R&D Systems, Inc., Minneapolis, MN). After rinsing with PBS, the sections were incubated with FITC-conjugated secondary antibody (Vector Labs, Burlingame, CA) for 60 minutes in 3% horse serum. For tracking EdU-positive cells, tissue sections were incubated with Click-IT reaction cocktail containing Alexa594-azide (Invitrogen) for 30 minutes at room temperature, then washed with 1 ml of 3% BSA in PBS. Nuclear staining was performed with 4,6-diamidino-2-phenylindole (DAPI, Invitrogen). The negative control experiments were also performed where the secondary antibodies or primary antibodies were omitted to exclude the potential non-specific binding to tissue specimen.

### Image analysis

The stained tissues were examined with Nikon Eclipse E600 fluorescence microscope and photographed with Retiga 1300 Q-imaging camera using the ACT-1 software (Nikon Instruments Inc., Melville, NY). Individual images generated from the green, red, and blue channels were superimposed to generate the composite figures. The images were then quantified using Image-Pro Plus image software (Media Cybernetics, Silver Spring, MD). For quantitative analysis of cells labeled by EdU or stained positive for a given cell marker, 5 renal tissue sections from each rat kidney were used. For each renal tissue section, 5 randomly selected fields in the entire cortex and 3 fields in the mid-, tip-, and base-region of papilla were examined separately, and the number of EdU+ cells was manually counted at ×100 magnification. If these cells also stained positive for a particular cell marker, they were also scored. The number of cells that expressed a given cell marker was divided by the number of EdU+ cells to derive the marker expression rate in EdU+ cells.

### Statistical analysis

Statistical analysis of quantitative data generated in above-described image analyses was performed with Prism 5 (GraphPad Software, Inc., San Diego, CA). The resulting data were expressed as mean ± standard deviation. One-way ANOVA was used to determine significance, which was set at *P*<0.05.

## Results

### Distribution of EdU-labeled retaining cells in the developing kidney

**Fig 1:**
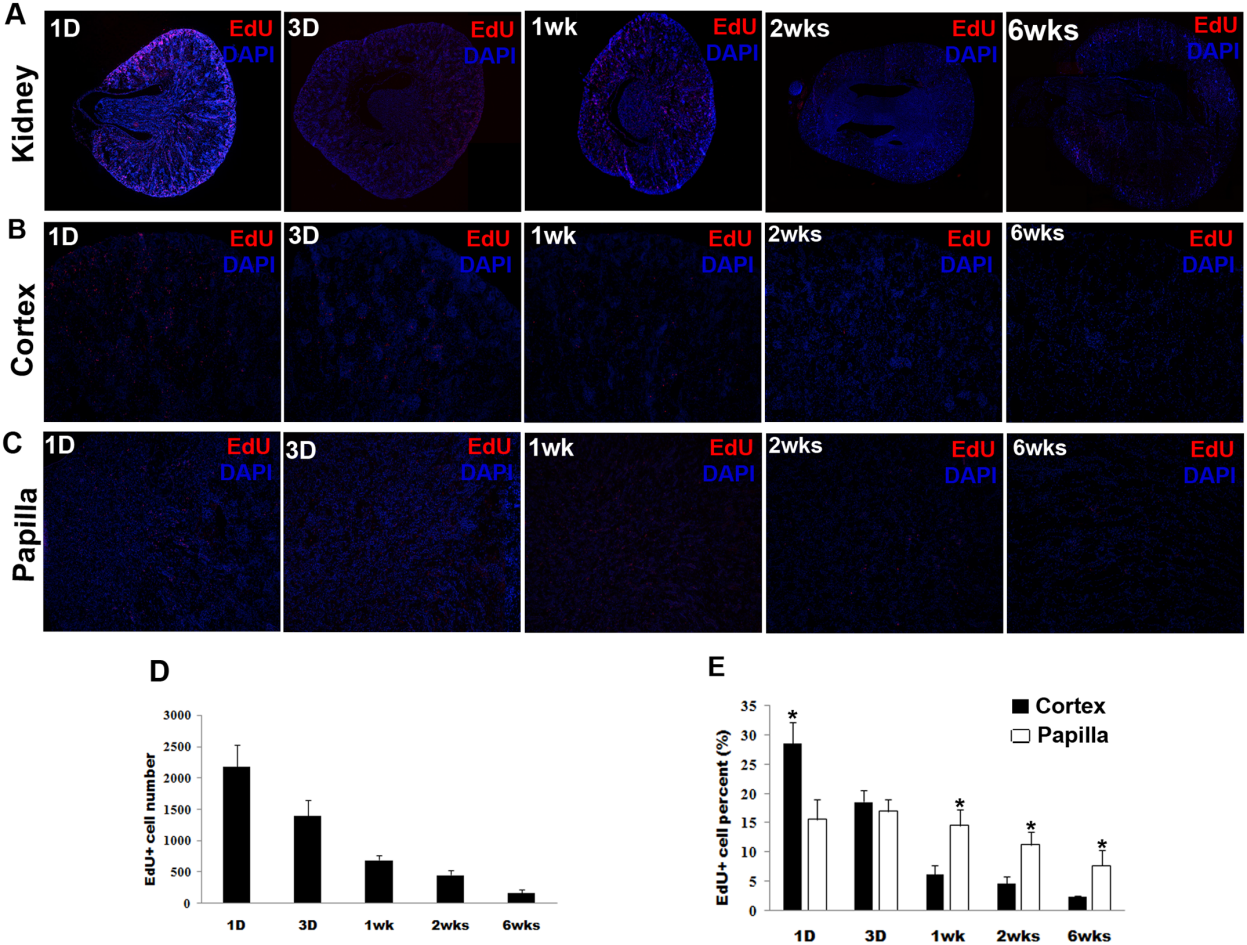
Differential EdU-labeled retaining in the developing kidney. Newborn rats received intraperitoneal injection of EdU. Their kidneys were harvested at 1 day, 3 days, 1 week, 2 weeks, and 6 weeks later and processed for staining for EdU with Alexa594-azide (red) and for cell nuclei with DAPI (blue). Five rats for each time point were used. The number of EdU+ cells was determined by counting the red stains at ×100 magnification in 5 renal tissue sections from each rat kidney. For each renal tissue section, 5 randomly selected fields in the entire cortex and 3 fields in the mid-, tip-, and base-region of papilla were examined separately. (A): The histologic distribution of EdU-labled cells in the entire kidney tissue (magnification: original at ×40); (B) and (C): The representative histologic images at different time points in the renal cortex and papilla (magnification: original at ×100); (D) The averaged numbers of EdU+ cells at each time point in the developing kidney; (E): The averaged EdU+ cell numbers were divided with the averaged total cell numbers to yield the values of EdU+ cell percentage shown in the bar chart. *P< 0.05 for comparisons between the cortex and papilla.

Intraperitoneal injection of EdU into newborn rats resulted in a high rate of EdU incorporation into kidney cells; but the labeled cells were lost progressively over time. At 1 day post-EdU injection, LRCs labeled by EdU were 2176.0 ± 355.6 cells in each renal tissue section, and dropped to 168 ± 48.4 cells by week 6 (Fig 1A and 1B). Moreover, as time increased, we found that the numbers of LRCs were differentially expressed in the renal cortex and papilla respectively. When EdU-labeled cells were counted separately between the cortex and papilla, the results were (per kidney section) 1278 ± 172.1 and 900 ± 216.1 in the cortex and papilla, respectively at day 1; and 11 ± 6.5 and 140 ± 48.9 in the cortex and papilla, respectively, at week 6. When these absolute numbers were converted into relative numbers (relative to the number of kidney cells per section), the results were 28.6 ± 3.6% and 15.6 ± 3.4% (*P*<0.05) in the cortex and papilla, respectively, at day 1; and 2.5 ± 0.1% and 7.7 ± 2.7% (*P*<0.05) in the cortex and papilla, respectively, at week 6 (Fig 1C–1E).

### Potential stem/progenitor cell markers are co-expressed with EdU+ cells in the glomeruli at different time points

**Fig 2:**
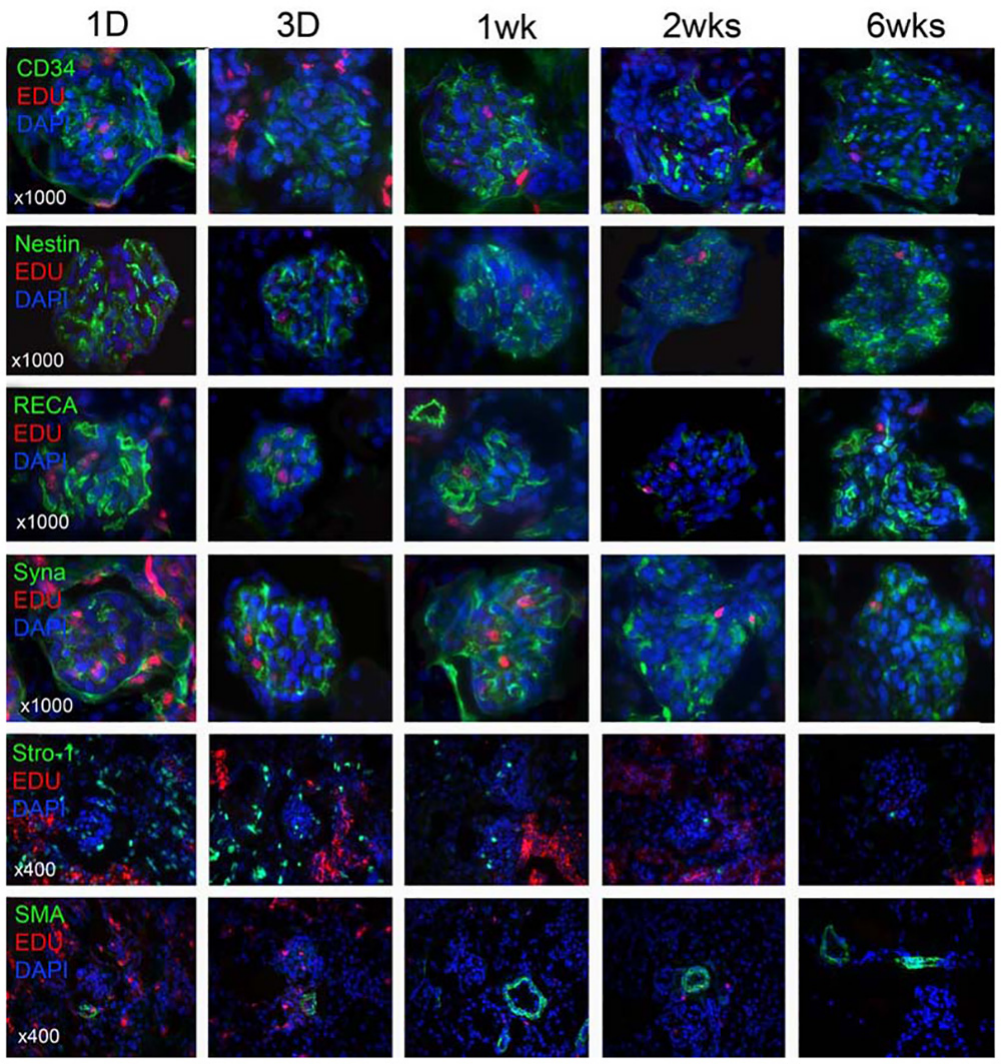
Co-localization of renal stem/progenitor cell marker and EdU in the glomeruli at different time points. Newborn rats received intraperitoneal injection of EdU. Their kidneys were harvested at 1 day, 3 days, 1 week, 2 weeks, and 6 weeks later and processed for staining with EdU (red), DAPI (blue) as well as the indicated progenitor cell marker (CD34, Nestin, RECA, Synaptopodin, Stro-1) (green). Representative histologic images at ×1000 and ×400 were shown stem/progenitor cell markers (upper 5 rows) and SMA (bottom row), respectively.

The glomeruli were the dominant location of EdU-labeled LRCs within cortex. Nestin, a widely employed marker of multipotent neural stem cell [13], was shown to be expressed in the glomeruli. Synaptopodin, an actin-associated protein that may play a role in renal podocyte foot processes [14], was also highly detectable in the glomeruli. Stro-1, serving as a mesenchymal stem cell marker [14], was present near the glomeruli. RECA, a protein essential for repair and maintenance of DNA [15], and CD34, a marker of endothelial and mesenchymal stem cells [16], were visible in the endothelium of glomeruli (Fig 2). No fluorescent signals of cell markers were detected in the control glomeruli samples where the secondary antibody had been omitted (S1 and S2 Figs).

**Fig 3:**
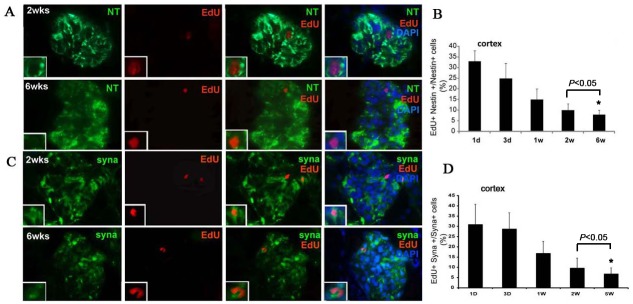
Co-localization of Nestin+/EdU+ and Synaptopodin+/EdU+ cells in the glomeruli at 2 and 6 weeks. (A) and (B): co-localization of Nestin+/ EdU+ in the glomeruli and ratio of cells positive for both Nestin and EdU among Nestin positive cells (n = 5, *P*<0.05); (C) and (D): co-localization of Synaptopodin+/ EdU+ in the glomeruli and ratio of cells positive for both Synaptopodin and EdU among Synaptopodin positive cells (n = 5, *P*<0.05). Magnification: original at ×1000.

Further analysis showed that the percentage of Nestin+/EdU+ double staining LRCs among Nestin positive cells was 33.3% ± 5.1% at day one post-injection, and dropped to 10.1 ± 2.0% at week 2 and to 8.3% ± 2.5% at week 6 post-injection in the glomeruli (Fig 3A and 3B). In addition, a small number of EdU-labeled LRCs also expressed positive for Synaptopodin in the glomeruli, but not in the papilla. These cells appeared irregular in shape and were detected with overlapped double stain. The ratio of cells positive for Synaptopodin+/EdU+ among Synaptopodin positive cells was 30.8% ± 10.2% at day one, and dropped to 9.8% ± 2.2% at week 2 and to 6.7% ± 3.4% at week 6 in the glomeruli (Fig 3C and 3D). A statistical significance was observed between the two time points of week 2 and week 6 (*P*<0.05).

### Differential cell marker expression and EdU-labeled LRCs in the renal cortex and papilla at 6-week time point

**Fig 4:**
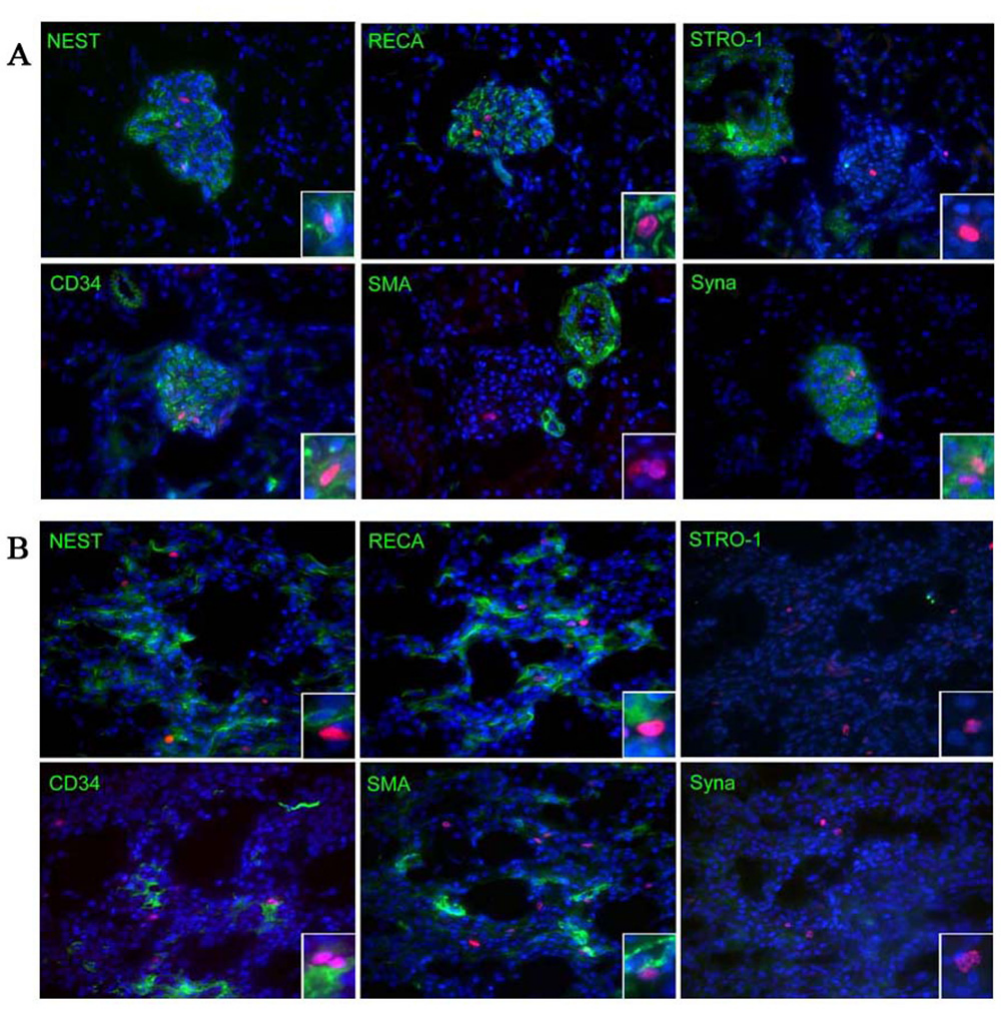
Differential cell marker expression and EdU-labeled LRCs co-localization in renal glomeruli and papilla at 6 weeks. (A) renal glomeruli; (B): renal papilla. The same tissue samples generated as described in Fig 1 were subject to immunofluorescence staining for the indicated cellular proteins (green stains). The resulting histological images were superimposed with images produced by EdU (red) and DAPI (blue) staining to produce the final three-color images. For the purpose of focusing on cell marker expression in the long term EdU-labeled LRCs, only the results of the 6-week tissue samples are shown here. The magnifications are 400× and 1000× for the panels and inserts, respectively.

Given that label retention in a very slow cycling tissue like the kidney is difficult to establish, it is likely that the EdU incorporation is a marker of proliferating cells rather than “label-retaining”, particularly at earlier postnatal time points. Therefore, in the current study, we focused on week 6 post-EdU injection to identify the potential renal progenitor cells. At 6 weeks postnatal time point, the long term EdU-labeled LRCs combined with expression of CD34, RECA-1, Nestin, and Synaptopodin were discretely but widely distributed within the glomeruli of renal cortex. Stro-1 was identified mostly around the glomeruli and SMA only in arterioles and arteries (Fig 4A). Up to approximately half of the EdU+ LRCs detected in the glomeruli are positive for Synaptopodin, Nestin, and CD34, and that nearly one-third are positive for RECA-1 (ranging from 31 to 56%), while markers that were detected outside of the glomeruli were expressed in LRCs at low rates such as Stro-1 and SMA (8 and 8.1%) (Table 1). Co-expression of CD34, RECA-1, and Nestin markers with the long term EdU-labeled LRCs was also identified only in the renal tubules of papilla (Fig 4B) but significantly lower than that in glomeruli (ranging from 2.5 to 4.8%, Table 1). Moreover, Stro-1 and Synaptopodin positive EdU-labeled LRCs were not detected in the renal tubules. In addition, in the control tubules samples at different time points omitted incubation with the primary antibodies, no fluorescent signals of Nestin were detected (S3 Fig), and similar results for other cell markers including CD34, RECA-1, SMA, Stro-1 and Synaptopodin.

**Table 1:** Differential expression of cell markers in the long term EdU-labeled LRCs in the renal cortex glomeruli and papilla tubules at 6 weeks.

Cell markers	Renal cortex glomeruli (%)	Renal papilla tubules (%)	*P* value*
**Nestin**	56 ± 7.9	2.5 ± 0.7	<0.01
**RECA**	31 ± 9.5	4.0 ± 1.2	<0.01
**CD34**	56 ± 7.9	4.8 ± 1.1	<0.01
**Synaptopodin**	50 ± 10.1	0.0 ± 0.0	<0.01
**STRO-1**	8.1 ± 1.5	0.0 ± 0.0	<0.01
**SMA**	8.0 ± 2.5	5.0 ± 1.4	0.10

*P value was calculated for comparisons between the cortex and papilla.

## Discussion

Several studies have employed the BrdU and its analogs to label slow cycling cells, a characteristic of stem cells and progenitor cells in the kidney, and the dynamic changes in LRCs identified by BrdU labeling have been shown in the renal papilla and tubules [7, 17–21]. The BrdU staining by antibody labeling is straightforward, however, the stem cell marker expression in BrdU-labeled cells are often difficult to detect because the complementary base pairing in double-stranded DNA blocks the access of the anti-BrdU antibody to BrdU subunits. Therefore, the present study introduced a new LRC procedure with the use of EdU to overcome the ambiguity, and examined the time-dependent distribution of EdU-labeled cells in the renal glomeruli and papilla tissues. Our data confirmed the prior work and showed that the kidney is undergoing substantial changes in development postnatally; particularly, a novelty of our study is the findings of label-retaining cells in the glomerulus.

In the present study, we determined the absolute and relative numbers of EdU-labeled cells at each of the 5 time points after intraperitoneal injection of EdU into newborn rats. We found that EdU was incorporated into the kidney at a high rate within the first day—approximately 22% of renal cells were labeled during this period. But as time progressed, the number of labeled cells also decreased sharply within 1 week. At 6 weeks post-EdU injection, only about 5% of renal cells remained labeled. These observations are consistent with most LRC studies and generally reflect the rapid cell cycling in most neonatal animal tissues [11, 22–24]. Interesting and unreported previously is that, initially (at day 1 post-injection) the cortex had a higher labeling rate than the papilla (28.6% vs. 15.5%), but by week 1 the cortex had fewer EdU+ cells than the papilla, and in the end (at week 6) the latter became the dominant site for LRCs (2.5% vs. 7.7%). Several studies have shown that the papilla may be one major niche for adult kidney stem cells [19,20,25]. Consistently, our quantitative kinetics data suggested that the asymmetrical differentiation and proliferation of label retaining cells may occur in the glomeruli and papilla tubules during the kidney development; however, the underlying mechanisms need to be explored further.

Also noteworthy is that at 6 weeks postnatal time point, the long term EdU-labled LRCs were found exclusively within glomeruli in the cortex, while in the papilla, LRCs were within and around tubules. These findings may be related to the development of the kidney. In the developing kidney, it has been reported that comma-shaped bodies and S-shaped bodies emerged at rat embryonic day 16 (E16), and renal corpuscles, composed of glomeruli and tubules, emerged at E18, nearly mature at postnatal day 7 (P7) accompany with the disappearance of comma-shaped and S-shaped cells. The number of the renal corpuscles increased by approximately 7 folds from E18 to P7 and achieved peak number at P7, while the volume of renal corpuscles increased approximately 86 folds from E18 to postnatal day 40 (P40, approximately 6 weeks) and no changes after P40 [26,27]. Given that label retention in a very slow cycling tissue like the kidney is difficult to establish, it is likely that the EdU incorporation is a marker of proliferating cells rather than “label-retaining”, particularly at earlier postnatal time points. However, we assessed the EdU-labeled cells profile throughout the postnatal development of rat kidney, which has considerable dynamics, to an adult morphological phase.

The localization of population of label-retaining cells may be different at each stage of kidney development. In our study, the time-dependent distribution of EdU-labled LRCs suggested that differences exist between cortex and papilla in the development of the kidneys. Of note, at the stage of EdU postnatal injection (immediately after birth) the rat kidneys are still developing, thus observation of EdU+ cells may represent remnants of the pool of embryonic progenitor cells in young adults. To further identify potential residing stem cells in the adult rat kidneys, more studies will be needed by injecting EdU at adulthood, e.g. week 5, and following for 6 weeks (or longer), and then analyzing the EdU-labled LRCs in adult kidneys. Maeshima et al. [18] reported the presence of BrdU label-retaining renal stem cells in 7-week-old rat renal tubules after BrdU injection for a 2-week chase, and these adult kidney tubular cell populations showed phenotypic plasticity, tubulogenic capacity, and integration capability into developing kidney. While in the other study by Oliver et al. [20], neonatal kidneys were labeled with BrdU for 3 days and were chased for 2 months, and the label-retaining cells were detected in the interstitium of renal papilla and contributed to kidney repair when injured.

The kidney is a specialized low-regenerative organ with several different types of cellular lineages. As known, unlike the embryonic stem cells possessing defined markers (Oct4, Nanog, etc), the adult or tissue-specific stem/progenitor cells are usually lack of identified single markers. In addition, it has become increasingly clear that very few markers are truly tissue-specific and, importantly, that cells of different origins can move in and out of domains of gene expression, altering their own expression pattern according to their current location [28]. To date, the markers that could possibly identify stem cells in the kidney differ among the published studies [17–21, 29]. In this study, we investigated 6 cell markers that related to undifferentiated podocytes, hematopoietic and mesenchymal stem cells, endothelial and smooth muscle cells, which may provide some clues to identify the potential renal stem/progenitor cells. They included Nestin (a marker of multipotent neural stem cell [13] which has been used in the search for renal stem cells [19,20]), Synaptopodin (an actin-associated protein [14] which may play a role in renal podocyte foot processes [30,31]), Stro-1 (a mesenchymal stem cell marker [14,32] which has been shown to express around the glomerulus in rat [33], RECA-1 (a protein essential for repair and maintenance of DNA which has been used to identify rat endothelial cells in various tissues [11,15]), CD34 (a marker of endothelial and mesenchymal stem cells [16]), and SMA (a smooth muscle actin which is useful for identifying blood vessels in non-smooth muscle organs such as the kidney).

At 6-week time point, in the renal cortex, Synaptopodin and Nestin were expressed discretely but widely in the glomerulus, as seen in previous studies [30,31]. CD34 and RECA-1 were found within the glomerulus, reflecting their association with the glomerular endothelium. Stro-1 was detected around the glomerulus, just as reported previously [32], and SMA was identified in larger blood vessels outside of the glomeruli. Interestingly, cellular markers that were detected in the glomeruli were expressed in EdU-labeled LRCs at high rates, such as Synaptopodin, Nestin, CD34, and RECA-1 (ranging from 31 to 56%), while markers that were detected outside of the glomeruli were expressed in LRCs at low rates, such as Stro-1 and SMA (8 and 8.1%). On the other hand, co-expression of CD34, RECA-1, and Nestin with the long term EdU-labeled LRCs was lower in renal papilla tubules (ranging from 2.5 to 4.8%) compared with the renal cortex glomeruli, while Stro-1 and Synaptopodin were not detected. These data suggested a different role of stem cells in the development of glomeruli and papilla tubules due to their complex structure, of which the mechanisms need to be clarified further.

Podocytes, the critical component of glomerular filtration barrier, cannot proliferate in adult kidney. However, recent experimental and clinical evidence have shown that glomerular injury can undergo regression under certain circumstances, suggesting that there may be one or more podocyte progenitors [34,35]. For example, Pippin et al reported that cells of renin lineage residing alongside glomerular capillaries have progenitor capacity to regenerate podocytes [36], but molecular mechanisms that generate podocytes are poorly understood [37]. Synaptopodin is an actin-associated protein that contributes to foot process formation in differentiated podocytes [14]; a recent renal stem cell study used its expression status to distinguish mature from immature podocytes [30]. Nestin is a cytoskeleton-associated class VI intermediate filament protein proposed to be a marker of multi-lineage progenitor cells [13], and has been used as a marker in the search for renal stem cells [19,20,38,39]. Several researchers have found that Nestin is normally expressed in podocytes [40–42], and in the present study we did not only confirm Nestin’s expression in the glomeruli but also identified its expression in renal tubules. Furthermore, we also found that Nestin was expressed at a high rate (~56%) in LRCs in the cortex, and this seems to be due to Nestin’s overall high expression rate in the glomeruli.

CD34 is a member of the sialomucin family and commonly regarded as a marker for hematopoietic stem cells (HSCs) [16]. Indeed, in two renal stem cell studies CD34 was used as an HSC marker in flow cytometric analysis of single-cell suspensions of rat and mouse kidneys, respectively [20,38]. However, in a recent review article we presented evidence that CD34 is also a marker for mesenchymal stem cells (MSCs) in all postnatal tissues and in freshly isolated MSCs [43].

On the other hand, it should be noted that stem cell markers in human cells may not indicate stemness in animal cells. For example, Prominin-1 (alias CD133), a neural and hematopoietic stem cell marker, was expressed in a limited fashion in human kidney but labeled the proximal tubule in mouse model [44]; and Sca-1, which is thought to label stem cells, particularly of the bone marrow, but in mouse kidney labeled the distal tubule [45]. Thus, although our current data reported the kinetics of EdU-retaining cells with potential stem markers in the development of rat kidney, stem cell character awaits further evaluation. More studies are required to confirm whether these LRCs were pluripotent and find out which one of these cell populations might be the true putative progenitor/stem cells by isolating or tracing the EdU^+^/nestin^+^ cells, EdU^+^/synaptopodin^+^ cells, and other EdU^+^/co-expressing markers from the papilla and glomeruli. In our previous study, we have employed the approach to identify the presence of potential stem cells in the rat penis by co-localizing EdU-retaining and cell markers [24]. These EdU-labeled penile corporal LRCs were further harvested and cultured in vitro, and were observed to possess the ability to form clones, giving a clue that the approach of combination of EdU-retaining and putative stem cell markers is useful to identify tissue-specific stem/progenitor cells.

Lineage tracing method offers a powerful tool to study cell fate and evaluate stem cell potency *in vivo*, particularly using ligand-inducible Cre recombinase (Cre) to trigger reporter expression at certain time points [46]. For example, Barker et al [47] showed that Lgr5-expressing cells within the S-shaped body are an intratubular progenitor population that gives rise to the thick ascending limb and distal convoluted tubule. Gomez et al [48] used a renin-Cre line to show that renin-expressing cells give rise to multiple lineages during development. In addition, Bussolati et al [49] identified a resident population with progenitor characteristics along the human nephron using the CD133 stem cell marker, which also exhibited the embryonic renal markers Pax-2, Six 1, and Six 2 and several mesenchymal stem cell markers. Although this powerful technique also has limitations, such as leakiness of the CreERt2, incomplete recombination and false-negative results, and interpretation of complex results, it is being increasingly applied to understand the mechanisms that govern kidney injury and regeneration [50,51].

The ability for tissue repair and regeneration necessary to reacquire functionality after ischemic, toxic, or inflammatory injury is limited in the adult kidney, and the contribution of stem/progenitor cell population to regeneration of the kidney in response to renal injury remains largely unknown and debated. Several animal studies have investigated that regeneration of injured renal tissues may occur through mechanisms that rely on the pre-existing resident progenitors able to survive injury, or the dedifferentiated resident cells to proliferate and substitute the damaged tissue, and the secretion of renoprotective or tropic factors [21, 52,53]. In this study, to find out which one of the EdU-retaining cell populations with potential cell markers might be the true stem/progenitor cells, needless to say, additional new techniques, such as *in vivo* cell lineage tracing method and renal ischemia/reperfusion injury animal model, are needed to confirm the stem cell fate, characteristics of clonogenicity and differentiation potency, and contributions to renal repair and regeneration when injured.

## Conclusions

Our interesting initial findings, for the first time, showed a co-expression of EdU-labeled LRCs with some known stem cell/kidney markers at different time points in the glomeruli and papilla tubules in the developing rat kidney. At 6 weeks post-injection time point, EdU-labeled LRCs existing in the glomeruli expressed undifferentiated podocyte and endothelial markers at high rates, while those existing in the renal tubules expressed Nestin and vascular markers at low rates. The EdU-LRC/cell markers strategy gave a clue to identify stem/progenitor cells in the kidney. However, to understand the characterization and localization of these EdU-LRCs, further studies will be needed to test cell lineage tracing, clonogenicity and differentiation potency, and the contributions to the regeneration of the kidney in response to renal injury/repair.

## Supporting Information

S1 Fig Co-localization of renal stem/progenitor cell marker and EdU in the glomeruli at 1 day post-injection. Newborn rats received intraperitoneal injection of EdU, and their kidneys were harvested at 1 day and processed for immunofluorescent staining (shown at ×400 of magnification). (A) Representative images of the glomeruli staining with EdU (red), DAPI (blue) as well as cell markers (Nestin, CD34, RECA, Synaptopodin, Stro-1, SMA) (green), respectively; (B) Representative images of the negative control sections of glomeruli omitted incubation with the secondary antibody but included all other steps. No fluorescent signals of cell markers (green) were observed in the control samples.
(TIF)

S2 Fig Co-localization of renal stem/progenitor cell marker and EdU in the glomeruli at 3 days post-injection. Newborn rats received intraperitoneal injection of EdU, and their kidneys were harvested at 3 days and processed for immunofluorescent staining (shown at ×400 of magnification). (A) and (B), the same as mentioned above in S1 Fig.
(TIF)

S3 Fig Co-localization of Nestin+/EdU+ cells in the renal tubules at different time points. Newborn rats received intraperitoneal injection of EdU. Their kidneys were harvested at 1 day, 3 days, 1 week, 2 weeks, and 6 weeks later and processed for staining. (A) Representative images of the tubules staining with EdU (red), DAPI (blue), and cell marker Nestin (green), at 1D、3D、1wk、2wks、6wks post-injection respectively (shown at ×400 of magnification); (B) Representative images of the control sections of tubules omitted incubation with the primary antibody but included all other steps. No fluorescent signals of cell markers (green) were observed in the control samples.
(TIF)
