# Supplementary figures and images for: Kinetics of Label Retaining Cells in the Developing Rat Kidneys
Source: PLoS One. 2015 Dec 9;10(12):e0144734. doi: 10.1371/journal.pone.0144734 (PMC4674088; doi:10.1371/journal.pone.0144734)

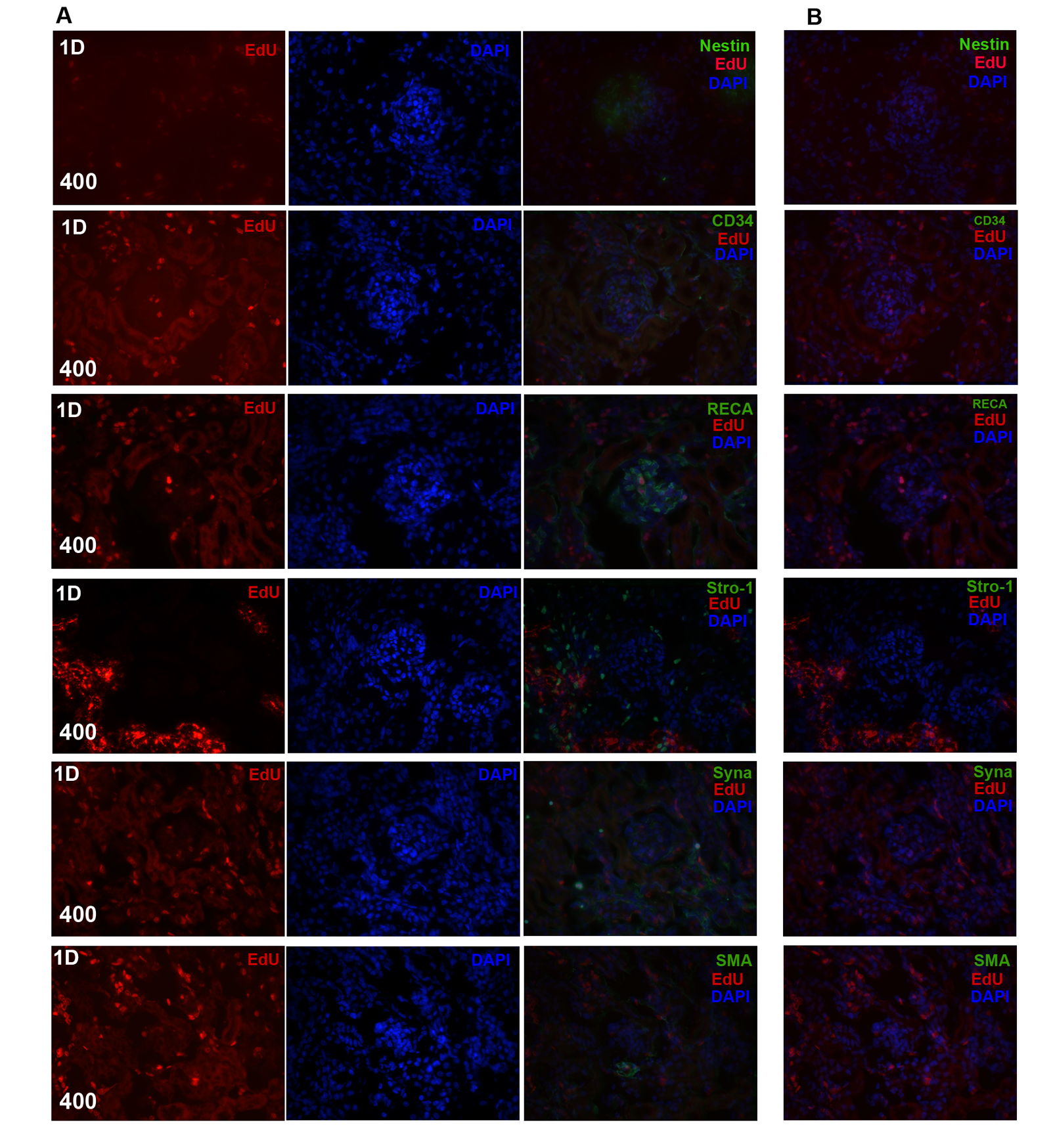

Supplement: S1 Fig — Newborn rats received intraperitoneal injection of EdU, and their kidneys were harvested at 1 day and processed for immunofluorescent staining (shown at ×400 of magnification). (A) Representative images of the glomeruli staining with EdU (red), DAPI (blue) as well as cell markers (Nestin, CD34, RECA, Synaptopodin, Stro-1, SMA) (green), respectively; (B) Representative images of the negative control sections of glomeruli omitted incubation with the secondary antibody but included all other steps. No fluorescent signals of cell markers (green) were observed in the control samples. (TIF) [file pone.0144734.s001.tif]

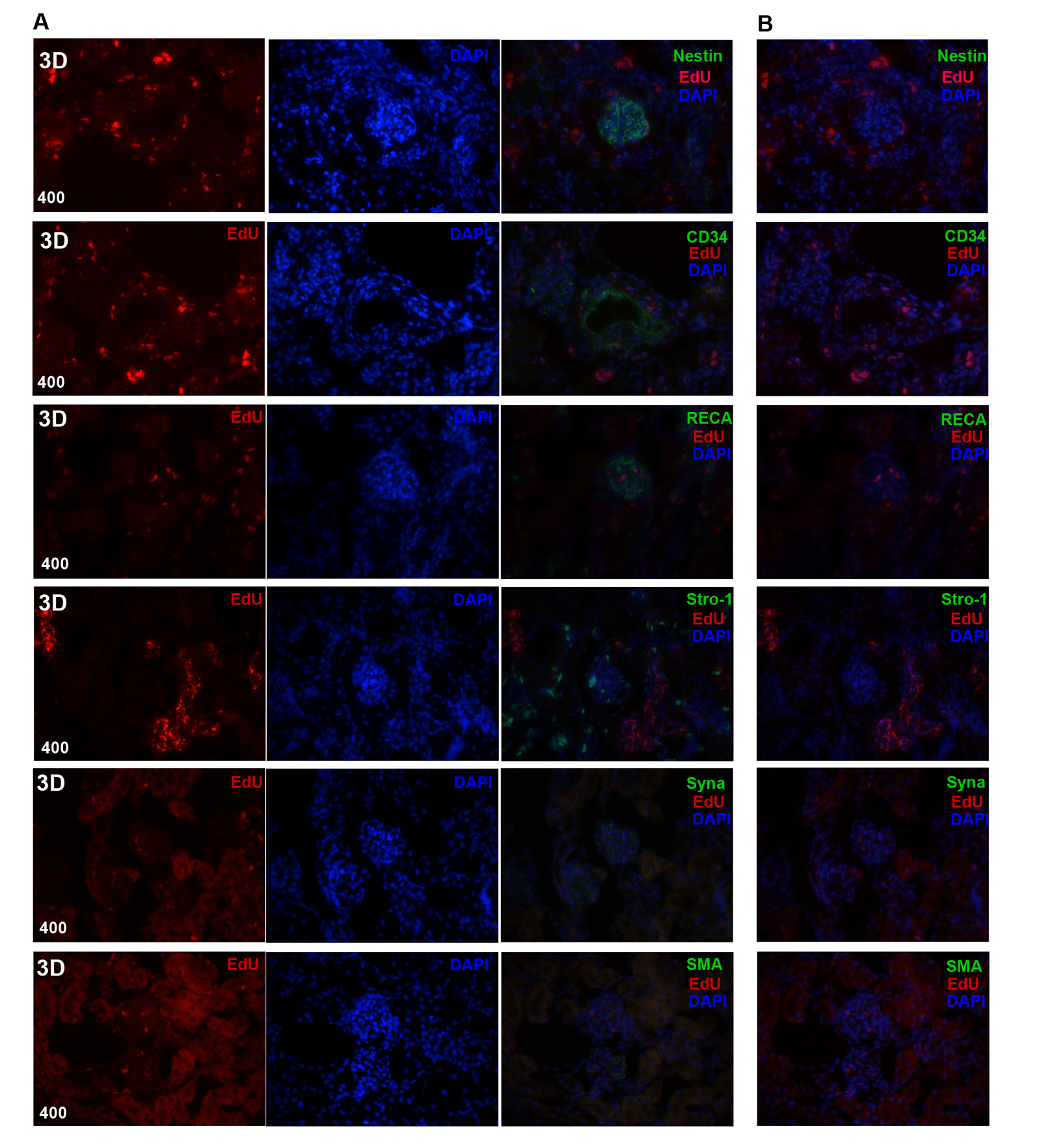

Supplement: S2 Fig — Newborn rats received intraperitoneal injection of EdU, and their kidneys were harvested at 3 days and processed for immunofluorescent staining (shown at ×400 of magnification). (A) and (B), the same as mentioned above in S1 Fig. (TIF) [file pone.0144734.s002.tif]

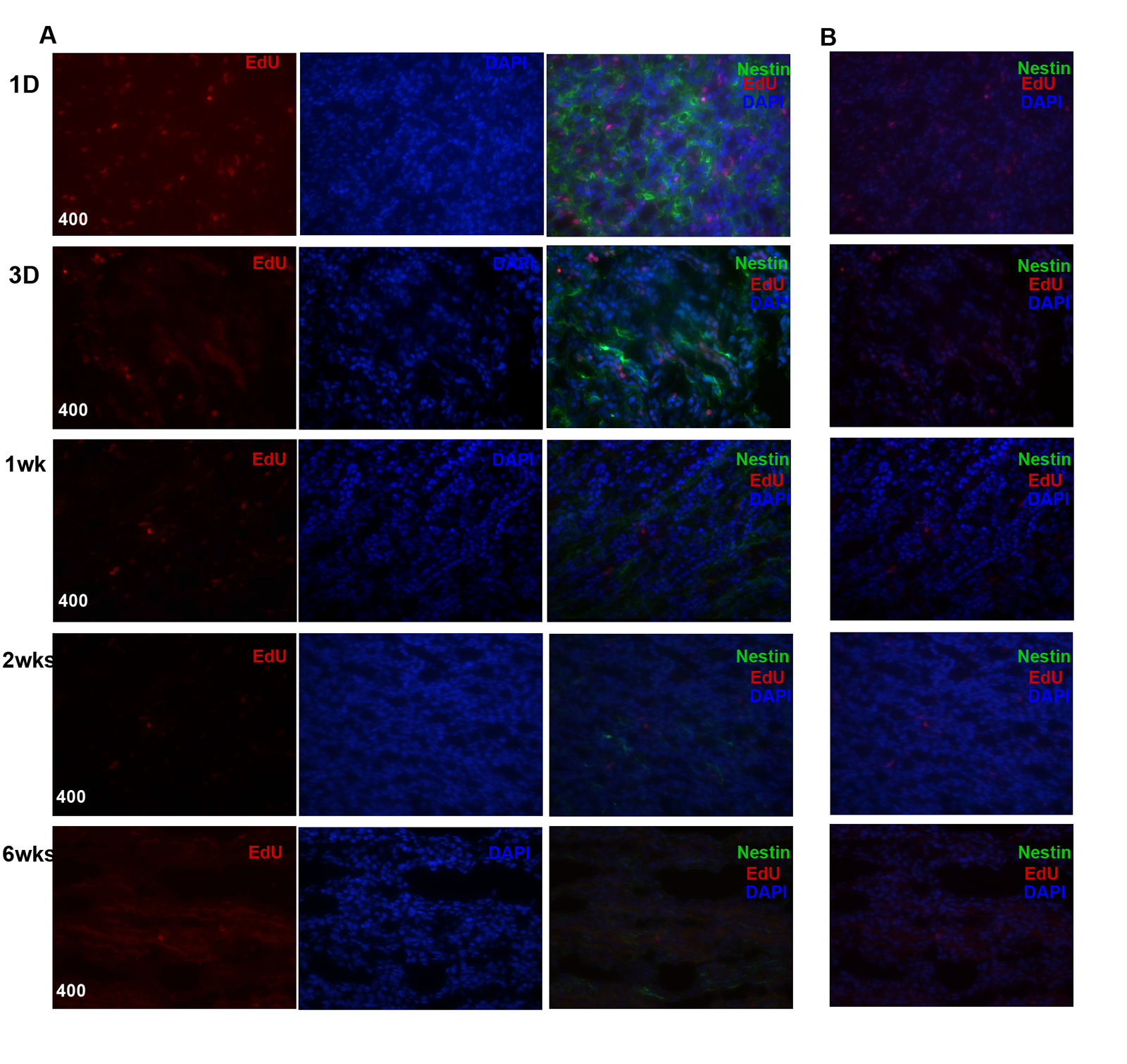

Supplement: S3 Fig — Newborn rats received intraperitoneal injection of EdU. Their kidneys were harvested at 1 day, 3 days, 1 week, 2 weeks, and 6 weeks later and processed for staining. (A) Representative images of the tubules staining with EdU (red), DAPI (blue), and cell marker Nestin (green), at 1D、3D、1wk、2wks、6wks post-injection respectively (shown at ×400 of magnification); (B) Representative images of the control sections of tubules omitted incubation with the primary antibody but included all other steps. No fluorescent signals of cell markers (green) were observed in the control samples. (TIF) [file pone.0144734.s003.tif]
